# Coordinated regulation of endocannabinoid-mediated retrograde synaptic suppression in the cerebellum by neuronal and astrocytic monoacylglycerol lipase

**DOI:** 10.1038/srep35829

**Published:** 2016-10-24

**Authors:** Xiaojie Liu, Yao Chen, Casey R. Vickstrom, Yan Li, Andreu Viader, Benjamin F. Cravatt, Qing-song Liu

**Affiliations:** 1 Department of Pharmacology and Toxicology, Medical College of Wisconsin, 8701 Watertown Plank Road, Milwaukee, WI 53226; 2 The Skaggs Institute for Chemical Biology, Department of Chemical Physiology, The Scripps Research Institute, 10550 North Torrey Pines Road, La Jolla, California 92037, USA

## Abstract

The endocannabinoid 2-arachidonoylglycerol (2-AG) mediates retrograde synaptic depression including depolarization-induced suppression of excitation (DSE) and inhibition (DSI). 2-AG is degraded primarily by monoacylglycerol lipase (MAGL), which is expressed in neurons and astrocytes. Using knockout mice in which MAGL is deleted globally or selectively in neurons or astrocytes, we investigated the relative contribution of neuronal and astrocytic MAGL to the termination of DSE and DSI in Purkinje cells (PCs) in cerebellar slices. We report that neuronal MAGL plays a predominant role in terminating DSE at climbing fiber (CF) to PC synapses, while both neuronal and astrocytic MAGL significantly contributes to the termination of DSE at parallel fiber (PF) to PC synapses and DSI at putative Stellate cell to PC synapses. Thus, DSE and DSI at different synapses is not uniformly affected by global and cell type-specific knockout of MAGL. Additionally, MAGL global knockout, but not cell type-specific knockout, caused tonic activation and partial desensitization of the CB_1_ receptor at PF-PC synapses. This tonic CB_1_ activation is mediated by 2-AG since it was blocked by the diacylglycerol lipase inhibitor DO34. Together, these results suggest that both neuronal and astrocytic MAGL contribute to 2-AG clearance and prevent CB_1_ receptor over-stimulation in the cerebellum.

The endocannabinoid 2-arachidonoylglycerol (2-AG) is arguably the best characterized retrograde messenger that provides feedback depression of synaptic transmission in the brain^1^,^2^. Depolarization-induced Ca^2+^ influx in postsynaptic neurons triggers the synthesis and release of 2-AG^3^,^4^, resulting in transient suppression of inhibition (DSI) or excitation (DSE)^5^,^6^,^7^. A brief tetanic stimulation induces synaptically evoked suppression of excitation (SSE) in the cerebellum, which is mediated by synaptic activation of the metabotropic glutamate receptor (mGluR1) and subsequent recruitment of 2-AG signaling^8^,^9^,^10^,^11^,^12^. 2-AG is degraded by serine hydrolases, primarily monoacylglycerol lipase (MAGL)^13^. Pharmacological inhibition or global genetic deletion of MAGL substantially prolonged DSE and SSE in the cerebellum and DSI in the hippocampus^14^,^15^,^16^,^17^, suggesting that hydrolysis of 2-AG by MAGL terminates endocannabinoid-mediated retrograde synaptic suppression.

Immunohistochemical studies have shown MAGL is not only expressed in neurons, but also in astrocytes^17^,^18^. In an attempt to determine the relative contribution of different brain cell types to 2-AG metabolism and signaling, we recently generated conditional knockout mice in which MAGL was deleted globally or specifically in neurons or astrocytes^18^. We showed that neuronal and astrocytic MAGL coordinately regulate brain 2-AG content, and cell type-specific deletion of MAGL from either neurons or astrocytes produces similar, modest but significant prolongation of DSE and SSE at the parallel fiber-Purkinje cell (PF-PC) synapses in the cerebellum, while global knockout of MAGL produces more dramatic prolongation of DSE and SSE^18^,^19^. In addition, we found that both neuronal and astrocytic MAGL limit the spread of endocannabinoid-mediated synaptic depression among PF-PC or PF-Golgi cell synapses and confer synapse-specificity of endocannabinoid signaling in the cerebellum^19^. Thus, both neuronal and astrocytic MAGL contribute similarly to the termination of retrograde 2-AG signaling at PF-PC synapses. However, it remains unknown whether cell type-specific knockout of MAGL alters DSE and DSI at other synapses in the cerebellum.

PCs, the single output neurons of the cerebellar cortex, receive excitatory inputs from PFs and climbing fibers (CFs)^20^,^21^. Depolarization of PCs induces DSE at both PF-PC and CF-PC synapses^6^. PCs receive inhibitory synaptic inputs from Basket cells (BCs) and Stellate cells (SCs), two types of interneurons that provide feed-forward inhibition to PCs^22^,^23^,^24^. DSI can be induced at interneuron-PC synapses^17^,^25^,^26^. MAGL global knockout prolonged DSI at SC-PC synapses but was not found to affect DSI at BC-PC synapses^17^. Thus, we studied the effects of cell type-specific MAGL knockout on putative SC-PC synapses.

It remains unknown whether cell type-specific knockout of MAGL affects DSE at CF-PC and DSI at SC-PC synapses. In the cerebellum, not all CB_1_R-positive nerve terminals express MAGL, and MAGL is not uniformly expressed in different cell types and cell layers^17^,^27^. Here, we sought to examine the effects of global and cell type-specific deletion of MAGL on DSE and DSI at several types of synapses onto PCs. In addition, we investigated the extent to which chronic increases in 2-AG content in MAGL knockout mice^18^ cause tonic activation and desensitization of CB_1_ receptors at PF-PC synapses. Our results revealed distinct effects of global and cell type-specific knockout of MAGL on endocannabinoid-mediated retrograde depression at different types of synapses.

## Results

### Effects of global and cell type-specific knockout of MAGL on DSE

**Figure 1:**
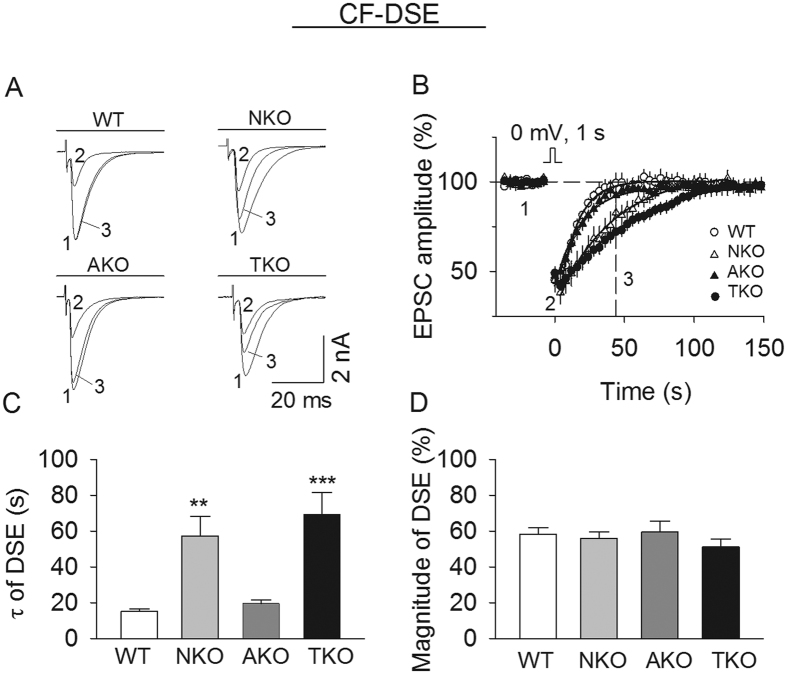
Effects of global and cell type-specific knockout of MAGL on CF-DSE in PCs. (**A,B**) Sample traces (**A**) and average time courses of CF-EPSCs (**B**) in response to a brief depolarization in cerebellar slices prepared from MAGL-TKO, -NKO, and -AKO mice and their wild-type (WT) littermates (n = 7–11 cells from N = 3–5 mice per genotype). The solid lines are single exponential fitting curves of the decay of CF-DSE, which yield the decay time constant (τ) of DSE shown in (**C**). (**C**) Compared with that of WT control, τ of CF-DSE was significantly increased in MAGL-TKO and -NKO mice but was not changed in MAGL-AKO mice (**p < 0.01, ***p < 0.001). (**D**) MAGL-TKO, -NKO and -AKO did not significantly alter the magnitude of CF-DSE (p > 0.05).

Depolarization of PCs induces DSE at both CF-PC and PF-PC synapses^6^. We first examined the extent to which global or cell type-specific knockout of MAGL altered DSE at CF-PC synapses in cerebellar slices. CF-EPSCs were evoked as described in Materials and Methods. One-way ANOVA showed that global knockout (MAGL-TKO) and neuron-specific knockout of MAGL (MAGL-NKO) substantially prolonged CF-DSE as indicated by the increases in the decay time constant (τ) (*F*_(*3,35*)_ = 11.45, *p* < 0.001; TKO *vs.* WT, *p* < 0.001; NKO *vs.* WT, *p* = 0.003), whereas astrocyte-specific deletion of MAGL (MAGL-AKO) did not significantly alter the duration of CF-DSE (*p* = 0.975, Fig. 1A–C). Global or cell type-specific knockout of MAGL did not significantly change the magnitude of CF-DSE (*F*_(*3,35*)_ = 1.15, *p* = 0.346; Fig. 1D). Thus, MAGL from neurons, but not astrocytes, plays a predominant role in terminating CF-DSE.

**Figure 2:**
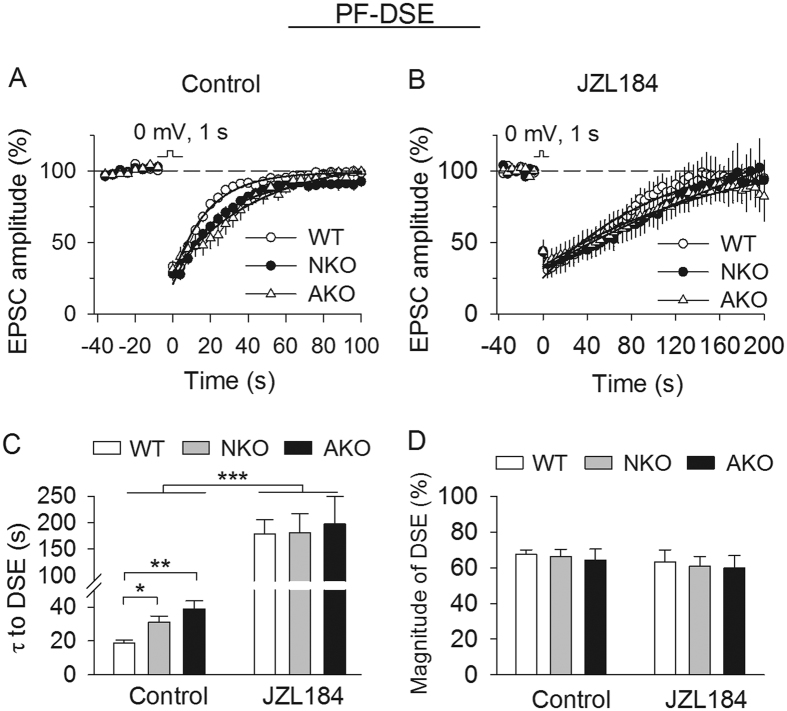
Effects of JZL184 on PF-DSE in cell type-specific MAGL knockout mice. (**A,B**) Average time courses of PF-DSE in MAGL-NKO, -AKO and WT slices (n = 7–9 cells from N = 3 mice per genotype) in the absence (control) or presence of JZL184. (**C**) τ of PF-DSE was significantly increased in MAGL-NKO and -AKO slices compared with that in WT slices (*p < 0.05, **p < 0.01). JZL184 significantly prolonged DSE in WT, MAGL-NKO and -AKO slices (***p < 0.001), and there were no differences in the τ of DSE among WT, MAGL-NKO and -AKO slices treated with JZL184 (*p* > 0.05). (**D**) The magnitude of PF-DSE was not significantly different in WT, MAGL-NKO and -AKO slices with or without JZL184 (*p* > 0.05).

The above results stand in contrast with findings obtained at PF-PC synapses, where neuronal and astrocytic MAGL contributes similarly to the termination of DSE and SSE^18^,^19^. The MAGL inhibitor JZL184 produces dramatic prolongation of DSE^14^,^28^ and SSE^19^, and does not further alter DSE at PF-PC synapses in slices prepared from MAGL global knockout mice^16^. SSE induced at PF-PC synapses in MAGL-TKO slices is not significantly different from that induced in wild-type slices treated with JZL184^19^. These results suggest that MAGL global knockout mimics and occludes the action of the MAGL inhibitor. However, it was unclear whether JZL184 exerts its effects by inhibiting neuronal MAGL, astrocytic MAGL, or both. In the present study, we determined whether JZL184 affected DSE at PF-PC synapses in MAGL cell type-specific knockout mice. Consistent with our previous study^18^, we found that MAGL-NKO and MAGL-AKO substantially prolonged PF-DSE compared with wild-type control (τ, *F*_(*2,23*)_ = 8.59, *p* = 0.002; NKO *vs.* WT, *p* = 0.037; AKO *vs.* WT, *p* = 0.002; Fig. 2A). Interestingly, JZL184 (0.3 μM) significantly prolonged DSE in wild-type, MAGL-NKO and MAGL-AKO slices (Fig. 2A,B) and abrogated the difference in the decay time constants (τ) of the three groups (*F*_(*2,23*)_ = 0.06, *p* = 0.938; Fig. 2C). The magnitude of DSE in these three groups was not significantly different in the absence (*F*_(*2,23*)_ = 0.13, *p* = 0.885) or the presence of JZL184 (*F*_(*2,23*)_ = 0.07, *p* = 0.931; Fig. 2D). Thus, JZL184 can inhibit either neuronal MAGL or astrocytic MAGL in MAGL-AKO and MAGL-NKO slices, respectively, to prolong DSE in MAGL cell type-specific knockout mice.

### Effects of global and cell type-specific knockout of MAGL on DSI

**Figure 3:**
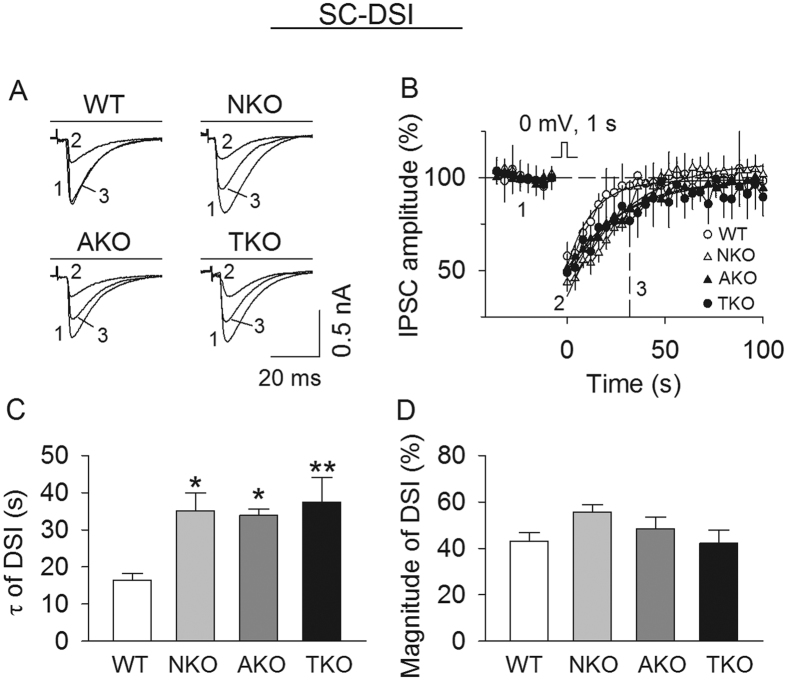
Effects of global and cell type-specific knockout of MAGL on DSI at putative SC-PC synapses. **(A,B**) Sample traces (**A**) and average time courses of IPSCs (**B**) in response to a brief depolarization in cerebellar slices prepared from MAGL-TKO, -NKO, and -AKO mice and their WT littermates (n = 10–12 cells from N = 3–4 mice per genotype). (**C**) τ of DSI was significantly increased in MAGL-TKO, -NKO and AKO mice compared to the WT control (*p < 0.05, **p < 0.01). (**D**) MAGL-TKO, -NKO and -AKO did not significantly alter the magnitude of DSI (*p* > 0.05).

SCs and BCs are GABAergic interneurons that receive excitatory inputs from PFs and provide feed-forward inhibition to PCs^22^,^23^. A brief depolarization of PCs could induce DSI at putative BC- and SC-PC synapses^17^,^25^,^26^. However, MAGL global knockout prolonged DSI at SC-PC synapses but had no effect on DSI at BC-PC synapses^17^. We therefore determined whether global and cell type-specific knockout of MAGL altered DSI at SC to PC synapses. The cell bodies of SCs are located in the outer molecular layer, whereas BC somata are found in the lower molecular layer just above Purkinje cells^29^,^30^. Putative SC-PC IPSCs were evoked by placing the stimulation electrode in the outer third of the molecular layer, which likely preferentially stimulates SC afferents^29^,^30^. However, we could not rule out a minor contribution of IPSCs from BC afferents in our studies. MAGL-TKO, -NKO and -AKO substantially prolonged DSI, as shown by significant increases in the decay time constant (τ) of DSI (*F*_(*3,40*)_ = 4.678, *p* = 0.007; TKO *vs.* WT, *p* = 0.009; NKO *vs.* WT, *p* = 0.028; AKO *vs.* WT, *p* = 0.045); the τ of DSI was not significantly different amongst the three groups of knockout mice (*p* > 0.05; Fig. 3A–C). The magnitude of DSI was not significantly changed amongst these groups (*F*_(*3,40*)_ = 1.906, *p* = 0.144; Fig. 3D). Thus, both neuronal and astrocytic MAGL contribute to the termination of DSI at putative SC-PC synapses.

### MAGL-TKO, but not MAGL-NKO or -AKO, caused tonic activation and partial desensitization of the CB_1_ receptor

**Figure 4:**
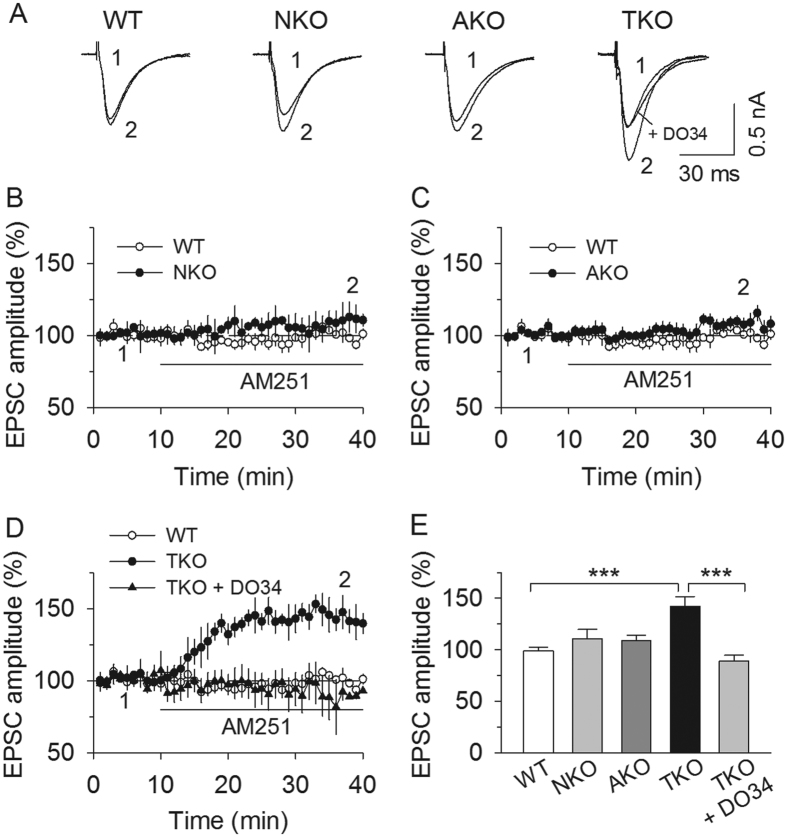
MAGL-TKO, but not -NKO or -AKO, caused tonic activation of the CB_1_ receptor. **(A–D**) Bath application of the CB_1_ receptor antagonist AM251 (2 μM) did not significantly alter PF-EPSCs in cerebellar PCs in MAGL-NKO (**A,B**; *p* > 0.05) and MAGL-AKO slices (**A,C**; *p* > 0.05) but caused a robust increase in the amplitude of PF-EPSCs in MAGL-TKO slices (**A,D**; *p* < 0.001, n = 7–9 cells from N = 3–4 mice per genotype). The AM251-induced increase in PF-EPSCs in MAGL-TKO slices was blocked by the DAGL inhibitor DO34 (**D**; *p* < 0.001). For the purpose of comparison, the result obtained from WT slices were superimposed in each panel from (**B–D**). (**E**) Summarized results show that bath application of AM251 induced a significant increase in PF-EPSCs in MAGL-TKO slices (***p < 0.001), but no significant change was found in MAGL-NKO and -AKO slices (*p* > 0.05). The increase in PF-EPSCs in MAGL-TKO slices was blocked by the DAGL inhibitor DO34 (***p < 0.001).

MAGL-TKO causes a drastic increase in 2-AG content in the brain, and MAGL-NKO or -AKO also cause a significant increase in 2-AG content^18^. If the increase in 2-AG content causes tonic activation of the CB_1_ receptor, CB_1_ receptor blockade should enhance basal synaptic transmission. To test this possibility, we examined the effects of the CB_1_ receptor antagonist AM251 on basal PF-EPSCs in PCs in MAGL global and cell type-specific knockout mice. Bath application of AM251 (2 μM) significantly increased PF-EPSCs in MAGL-TKO slices (*F*_(*3,32*)_ = 9.77, *p* < 0.001; TKO *vs.* WT, *p* < 0.001; Fig. 4A,D,E), but had no significant effects on PF-EPSCs in WT, MAGL-NKO and -AKO mice (NKO *vs.* WT, *p* = 0.581; AKO *vs.* WT, *p* = 0.746, Fig. 4A,B,C,E). These results revealed that global deletion of MAGL, but not cell type-specific deletion of MAGL causes persistent suppression of PF-EPSCs through tonic activation of the CB_1_ receptor.

We determined whether 2-AG mediated the tonic activation of the CB_1_ receptor in MAGL-TKO slices. 2-AG is synthesized by diacylglycerol lipase (DAGL), which converts diacylglycerol into 2-AG^31^,^32^. We have shown that the selective DAGL inhibitor DO34 was effective in blocking DSE and DSI in the cerebellum and hippocampus^33^. In MAGL-TKO slices treated with DO34 (1 μM), bath application of AM251 (2 μM) failed to alter PF-EPSCs (TKO *vs.* TKO + DO34, *t*_*13*_ = 7. 88, *p* < 0.001; Fig. 4A,D,E). Thus, 2-AG mediates tonic activation of the CB_1_ receptor in MAGL-TKO mice.

**Figure 5:**
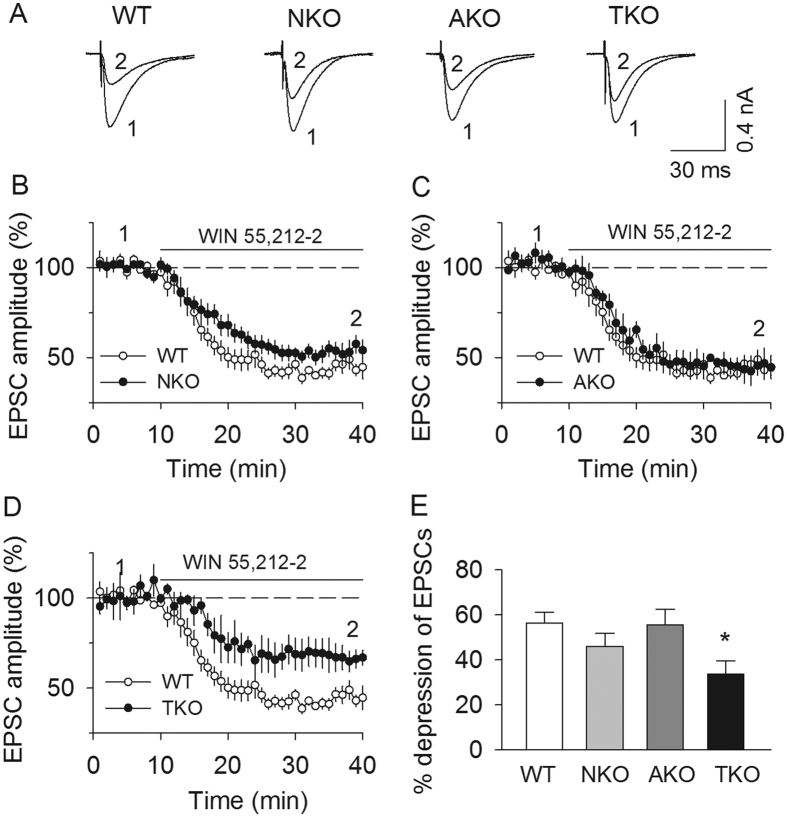
MAGL-TKO, but not -NKO or -AKO, caused partial desensitization of the CB_1_ receptor. **(A–D**) There was no significant difference in WIN55,212-2-induced PF-EPSC depression between WT and MAGL-NKO (**A,B**; *p* > 0.05), or between WT and MAGL-AKO slices (**A,C**; *p* > 0.05). However, WIN55212-2 induced significantly less depression of PF-EPSCs in MAGL-TKO slices compared with that of WT (**A,D**; *p = 0.045). For the purpose of comparison, the result obtained from WT slices were superimposed in each panel from (**B**–**D**). (**E**) Summarized results show that WIN55,212-2 induced a significantly smaller depression of PF-EPSCs in MAGL-TKO slices (*p = 0.045) compared with that of WT, but no a significant difference was found between MAGL-NKO vs. WT; or MAGL-AKO vs. WT (*p* > 0.05, n = 9–12 cells from N = 3–4 mice per genotype).

To test possible CB_1_ receptor desensitization, we examined whether the CB_1_ receptor agonist WIN55,212-2-induced depression of EPSCs was altered in MAGL global and cell type-specific knockout mice. Bath application of a saturating concentration (2 μM) of the CB_1_ agonist WIN55,212-2 produced significantly less depression of PF-EPSCs in cerebellar PCs in MAGL-TKO slices (*F*_(*3,41*)_ = 3.23, *p* = 0.033; TKO *vs.* WT, *p* = 0.045; Fig. 5A,D,E), whereas there was no significant difference in WIN55,212-2-induced depression of PF-EPSCs among wild-type, MAGL-NKO and -AKO slices (NKO *vs.* WT, *p* = 0.589; AKO *vs.* WT, *p* = 0.998, Fig. 5A,B,C,E). These results indicate that only global deletion of MAGL causes tonic activation and partial desensitization of the CB_1_ receptor at PF-PC synapses.

## Discussion

The endocannabinoid 2-AG mediates multiple forms of retrograde synaptic depression including DSE and DSI^3^,^4^,^34^. 2-AG is hydrolyzed primarily by MAGL^13^, which is expressed in both neurons and astrocytes^17^. The present study examined the effects of global and cell type-specific knockout of MAGL on DSE and DSI in the cerebellum. The results indicate that the impact of global and cell type-specific knockout of MAGL on DSE and DSI varied among different synapses. Both neuronal and astrocytic MAGL significantly contributed to the termination of PF-DSE and putative SC-DSI, while neuronal MAGL plays a predominant role in terminating CF-DSE.

PCs, the principal output neurons of the cerebellar cortex, receive excitatory synaptic inputs from PFs from granule cells and CFs from the inferior olive^20^. Both PF-PC and CF-PC synapses display DSE^6^. Pharmacological inhibition and global knockout of MAGL substantially prolongs DSE at both PF-PC and CF-PC synapses in cerebellar slices^14^,^16^,^17^. Global knockout of MAGL dramatically prolongs DSE and SSE at PF-PC synapses^16^,^18^,^19^, while neuron- and astrocyte-specific deletion of MAGL produces modest but significant prolongation of DSE and SSE^18^,^19^. Further, viral transduction of Bergmann glia (BG) with MAGL in a MAGL global knockout background significantly shortens PF-DSE^17^. We extended these studies in an important way by examining the effects of cell type-specific knockout of MAGL on DSE at CF-PC synapses, and for the purpose of comparison, on DSE at PF-PC synapses as well. Here we show that MAGL-NKO and -AKO produced similar, modest prolongation of PF-DSE. In contrast, MAGL-AKO did not significantly prolong CF-DSE, whereas MAGL-NKO and -TKO produced robust prolongation of CF-DSE. Thus, neuronal and astrocytic MAGL collaborate to terminate PF-DSE, whereas neuronal MAGL plays a predominant role in terminating CF-DSE.

Previous studies have shown that the MAGL inhibitor JZL184 produces dramatic prolongation of DSE and SSE at PF-PC synapses in cerebellar slices prepared from wild-type mice^14^,^19^. JZL184 does not further alter DSE at PF-PC synapses in slices prepared from MAGL global knockout mice^16^. We found that JZL184 significantly prolonged PF-DSE in wild-type, MAGL-NKO and MAGL-AKO slices, and there was no significant difference in the observed DSE among the three groups with JZL184. These results revealed that JZL184 can inhibit neuronal or astrocytic MAGL in MAGL-AKO and MAGL-NKO mice, respectively, to prolong PF-DSE.

Given that not all CB_1_R-positive nerve terminals express MAGL^17^, one may suspect that the impact of cell type-specific MAGL knockout on DSE is determined by selective distribution of MAGL at individual synapses. However, this appears to not always be the case. Immunohistochemical studies have shown that MAGL is highly enriched in the PF terminals in the molecular layer but is absent in CF terminals^17^. However, the CF terminals are surrounded by dense PF terminals. The degradation of 2-AG by MAGL expressed at heterosynaptic sites may explain why neuron-specific knockout of MAGL substantially prolongs DSE at CF-PC synapses. BGs are astrocytes in the cerebellum that have their cell bodies in the PC layer and processes that extend into the molecular layer^35^. MAGL is weakly expressed in the BG^17^. Both the CF and PF terminals are surrounded by processes of BGs. However, we found that astrocyte-specific knockout of MAGL substantially prolonged PF-DSE but did not have a significant effect on CF-DSE. It is unclear why astrocyte-specific deletion of MAGL produced different effects on PF- and CF-DSE.

It was not previously known whether MAGL cell type-specific knockout alters DSI in the cerebellum. We found that MAGL-TKO, -NKO and -AKO significantly prolonged DSI at putative SC-PC synapses. The stimulating electrode was restricted to the outer third of the molecular layer, which contains numerous GABAergic axonal terminals from SCs, the only neurons localized exclusively in the molecular layer^30^. However, the molecular layer does contain sparse axonal projections from BCs, the somata of which are located just above the PC layer^30^. There is a possibility that BC afferents make a minor contribution to IPSCs recorded in the present study. SCs provide feed-forward inhibition that temporally limits excitatory input to PCs and reduces spike generation by asynchronous inputs^22^,^24^. Immunofluorescent staining revealed that GABAergic nerve terminals in the molecular layer do not express MAGL^17^. However, GABAergic axonal terminals are surrounded by a dense network of PF terminals and BG processes that express MAGL, which may explain why neuronal and astrocytic deletion altered DSI at SC-PC synapses.

Previous studies have shown that genetic deletion or chronic blockade of MAGL caused elevations in 2-AG levels and CB_1_ receptor desensitization^16^,^36^,^37^. We found that, compared with that of wild-type mice, the magnitude of WIN55,212-2-induced depression of PF-EPSCs was decreased in MAGL-TKO mice, but not in MAGL-AKO and MAGL-NKO mice. Thus global knockout of MAGL, but not cell type-specific knockout of MAGL, causes partial CB_1_ receptor desensitization. This desensitization in MAGL-TKO mice is likely caused by 2-AG-induced tonic activation of CB_1_ receptors. Indeed, we found that AM251 significantly increased basal PF-EPSCs in MAGL-TKO mice, and the effect of AM251 was blocked by the DAGL inhibitor DO34^33^. There was no detectable tonic activation of CB1 receptors in MAGL-NKO and MAGL-AKO mice. These findings may reflect the sufficiency of the remaining neuronal or astrocytic MAGL in MAGL-AKO or -NKO mice, respectively, to clear 2-AG from the vicinity of CB_1_ receptors. These results support the idea that neuronal or astrocytic MAGL can clear 2-AG sufficiently to prevent over-stimulation and desensitization of CB_1_ receptors, as only global knockout of MAGL causes tonic activation and partial desensitization of the CB_1_ receptor.

Previously, CB_1_ receptor desensitization was investigated in global and cell type-specific MAGL knockouts using both behavioral and biochemical assays^18^. In these experiments, it was found that global knockout but not cell type-specific knockout was associated with behavioral and biochemical desensitization to the CB_1_ receptor agonist WIN55,212-2^18^. Our experiments are consistent with these findings, as only MAGL-TKO mice displayed evidence for tonic activation and desensitization of the CB_1_ receptor. Thus, our results add electrophysiological evidence in support of CB_1_ receptor desensitization in MAGL global knockout mice. However, the molecular mechanism underlying these findings remains unclear. As mentioned previously, CB_1_ receptor desensitization likely arises from chronically elevated 2-AG content resulting in CB_1_ receptor overstimulation. Indeed, chronic administration of cannabis or other CB_1_ receptor agonists in mice and humans leads to tolerance to several of the effects of these agonists and an associated CB_1_ receptor downregulation^38^,^39^,^40^,^41^,^42^,^43^,^44^,^45^,^46^, which may account for the partial desensitization seen in our studies.

MAGL accounts for about 85% of 2-AG hydrolysis in mouse brains. Additional serine hydrolases, including serine hydrolase alpha-beta-hydrolase domain 6 and 12 (ABHD6 and ABHD12), are responsible for the remaining 15%^13^. Selective ABHD6 inhibitors WWL123 and WWL70 did not alter DSE and DSI in cerebellar PCs in wild-type and MAGL global knockout mice^16^,^17^. Thus, ABHD6 likely does not play a significant role in 2-AG hydrolysis in the cerebellum, even in the context of MAGL global knockout. It remains to be determined whether ABHD12 contributes to the termination of DSE and DSI. However, this cannot be investigated until a specific ABHD12 inhibitor becomes available.

In summary, we have found that in most synaptic inputs to PCs, both neuronal and astrocytic MAGL contributed significantly to the termination of DSE and DSI. Neuronal MAGL, but not astrocytic MAGL, significantly contributed to the termination of CF-DSE. In addition, we found that MAGL-TKO, but not -NKO and -AKO, caused tonic activation and partial desensitization of CB_1_ receptors at PF-PC synapses. These results suggest that neuronal and astrocytic MAGL collaborate to terminate endocannabinoid-mediated synaptic suppression at most synapses.

## Material and Methods

### Animals

All animal use was in accordance with protocols approved by the Institution’s Animal Care and Use Committee of Medical College of Wisconsin. Wild-type (WT) and MAGL global and cell type-specific knockout mice were generated as described in our previous studies^18^. Total MAGL knockout (MAGL-TKO) and wild-type littermates were generated by breeding homozygous *Mgll*^*loxP/loxP*^ mice with Rosa26-Cre^47^. Neuron-specific (MAGL-NKO) and astrocyte-specific (MAGL-AKO) were generated by crossing *Mgll*^*loxP/loxP*^ to Eno2-Cre^48^ and GFAP-Cre^49^ mice respectively, then backcrossing the resulting double heterozygotes (*Cre*^+/−^*, Mgll*^+*/loxP*^) to *Mgll*^*loxP/loxP*^ to produce cell type-specific MAGL knockout mice (*Cre*^+/−^*, Mgll*^*loxP/loxP*^) and wild-type littermates (*Cre*^−*/*−^*, Mgll*^*loxP/loxP*^). Although the GFAP-Cre line shows low-level (~2–6%) recombination in neurons^18^,^50^, concerns over Cre expression in neurons in MAGL-AKO mice are minimized by previous membrane proteome studies showing that neurons isolated from MAGL-AKO mice have normal 2-AG hydrolyzing capacity^18^. Genotyping was carried out by PCR using DNA sample obtained from the tail or ear^18^.

### Slice preparation

MAGL conditional knockout mice and wild-type littermates of either sex (11–18 days old) were anaesthetized by isoflurane inhalation and decapitated. The mouse brain was embedded in low-melting-point agarose, and parasagittal cerebellar slices (200–250 μm thick) were cut using a vibrating slicer (Leica VT1200s). Slices were prepared at 4–6 °C in a choline-based solution as described in our recent study^19^. Slices were allowed to recover for at least 1 hour in the artificial cerebrospinal fluid (ACSF) containing (in mM): 119 NaCl, 2.5 KCl, 2.5 CaCl_2_, 1 MgCl_2_, 1.25 NaH_2_PO_4_, 26 NaHCO_3_, and 10 glucose.

### Electrophysiology

Whole-cell voltage-clamp recordings were made from cerebellar PCs. Cells were visualized using infrared-differential interference contrast optics (Nikon Eclipse FN1 and Olympus BX51WI) and 40x water immersion lens. CFs were stimulated with a bipolar tungsten stimulation electrode (WPI) placed in granular layer; CF-PC EPSCs showed all or none responses at near threshold stimulation and exhibited strong paired-pulse depression^6^. PFs were stimulated with the stimulation electrode placed in the molecular layer. GABA_A_ receptor blocker picrotoxin (50 μM) was present in the ACSF throughout the recording of EPSCs. AMPA receptor antagonist CNQX (1–2 μM) was included in the ACSF to reduce the amplitude of CF-PC EPSCs. Inhibitory postsynaptic currents (IPSCs) were evoked by placing the stimulation electrode in the outer third of the molecular layer. SCs, the GABAergic interneurons in the cerebellar cortex, are localized in this area^23^. AMPA receptor antagonist CNQX (20 μM) and NMDA receptor antagonist CPP (2 μM) were present in the ACSF throughout the recording of putative SC-PC IPSCs. To induce DSE or DSI, PCs were depolarized from −70 mV to 0 mV for 1 s, and EPSCs or IPSCs were evoked at 4 s intervals while the cells were voltage-clamped at −70 mV.

Glass pipettes (2–3 MΩ) were filled with one of the following internal solutions containing (in mM): (1) 130 cesium methanesulfonate, 10 CsCl, 2 QX-314, 10 HEPES, 0.2 EGTA, 2 MgCl_2_, 4 Mg-ATP, 0.3 Na_2_GTP, and 10 Na_2_-phosphocreatine (pH 7.3 with CsOH) for examining DSE and the effects of WIN55,212-2 or AM251 on EPSCs; (2) 90 cesium methanesulfonate, 50 CsCl, 2 QX-314, 10 HEPES, 0.2 EGTA, 2 MgCl_2_, 4 Mg-ATP, 0.3 Na_2_GTP, and 10 Na_2_-phosphocreatine (pH 7.3 with CsOH) for examining DSI.

### Chemicals

Unless specified otherwise, all drugs were prepared as concentrated stock solutions and stored at −20 °C before use. Picrotoxin and 6-Cyano-7-nitroquinoxaline-2,3-dione disodium salt hydrate (CNQX) were purchased from Sigma-Aldrich, Inc. (RS)-3-(2-Carboxypiperazin-4-yl) -propyl-1-phosphonic acid ((*RS*)-CPP), (R)-(+)-[2,3-Dihydro-5-methyl-3-(4-morpholinylmethyl) pyrrolo[1,2,3-de]-1,4-benzoxazin-6-yl]-1-naphthalenylmethanone mesylate (WIN55,212-2 mesylate) and N-(Piperidin-1-yl)-5-(4-iodophenyl)-1-(2,4-dichlorophenyl)-4-methyl-1H-pyrazole -3-carboxamide (AM251) were purchased from Tocris Bioscience. MAGL inhibitor JZL184^51^ and DAGL inhibitors DO34^33^ was synthesized in the laboratory of Benjamin Cravatt at the Scripps Research Institute, USA.

### Drug application

JZL184 and DO34 were dissolved in DMSO as 4 or 10 mM stock solutions and stored at −20 °C. The stock solutions were diluted in the ACSF and the final concentration of DMSO in the ACSF was ≤0.2%. Slices were placed on a nylon net and were incubated with JZL184 or DO34 for 40 min before transferring into the recording chamber. Electrophysiological recordings were performed in the presence of the same concentration of inhibitors in the ACSF, and each experiment was terminated within 60 min after transferring slices to the recording chamber. AM251 and WIN55,212-2 were directly applied into the ACSF without pre-incubation.

### Data Analysis and Statistics

The EPSC and IPSC amplitudes were normalized to the baseline. The decay time constant (τ) and magnitude of DSE and DSI were measured as described previously^14^. The change (%) of EPSCs by the CB_1_ agonist/antagonist was calculated as follows: 100 × [mean amplitude of EPSCs at last 5 min of drug application/mean amplitude of baseline EPSCs]. Data are presented as the mean ± SEM. Results were analyzed with one-way ANOVA or Student’s t-test. Results were considered to be significant at *p* < 0.05.

## Additional Information

**How to cite this article**: Liu, X. *et al*. Coordinated regulation of endocannabinoid-mediated retrograde synaptic suppression in the cerebellum by neuronal and astrocytic monoacylglycerol lipase. *Sci. Rep.* **6**, 35829; doi: 10.1038/srep35829 (2016).
